# Supplementary material for: Segregation patterns in rotating cylinders determined by the size difference, density ratio, and cylinder diameter
Source: Sci Rep. 2023 Aug 18;13:13495. doi: 10.1038/s41598-023-40774-6 (PMC10439168; doi:10.1038/s41598-023-40774-6)
Supplement: Supplementary file 1 — Supplementary Information. [file 41598_2023_40774_MOESM1_ESM.pdf]

Supplementary Information for
“Segregation patterns in rotating cylinders determined by the size difference, density ratio, and cylinder diameter”

Kurumi Kondo, Hiroyuki Ebata, and Shio Inagaki

Department of Physics, Kyushu University, Fukuoka, 819-0395, Japan

A. Dependence of phase diagrams on the rotational speed

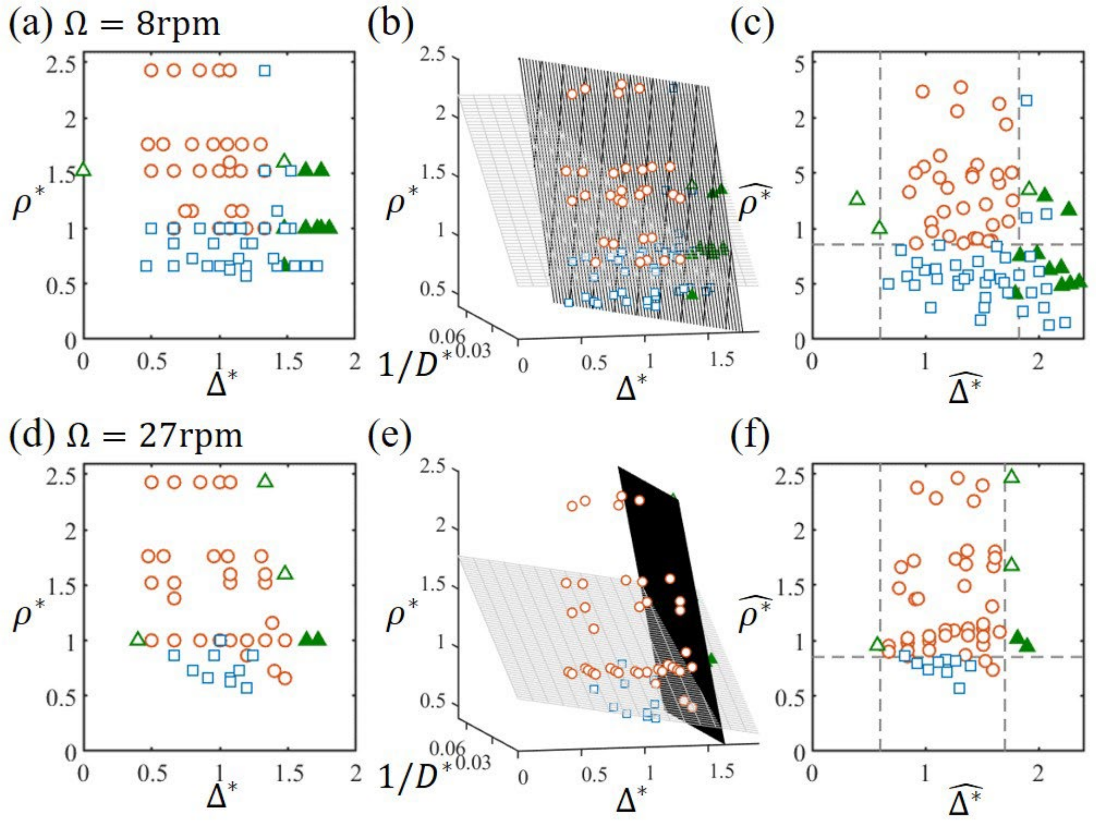

Figure S1. Phase diagrams of the segregated states: radial (blue squares), mixed ($\Delta^* > 0.5$, green filled triangle), mixed ($\Delta^* < 0.5$, green open triangle), and axial (red circles). Mixed states with $\Delta^* < 0.5$ and $\Delta^* > 0.5$ are distinguished and marked differently, reflecting their distinct origins of mixing. (a-c) $\Omega = 8$ rpm (d-f) $\Omega = 27$ rpm (a, d) Phase diagram using $\Delta^* = (d_l - d_s)/d_{av}$ and $\rho^* = \rho_l/\rho_s$. (b, e) 3D phase diagram using Δ^*, ρ^*, and $1/D^*$. The mesh planes indicate the separating planes. For visibility, the mixed state at $\Delta^* < 0.5$ is not plotted. (c, f) Phase diagram projected onto 2D space, with $\widehat{\Delta}^*$ and $\hat{\rho}^*$ as axes, according to Eqs. (1) and (2). The dotted lines correspond to the boundaries of the segregated state. The combinations of the particle species are chosen from glass, alumina, zircon, and zirconia beads, with diameters ranging from 0.2 mm to 4 mm. Three different inner cylinder diameters (D) are used: 74 mm, 54 mm, and 36 mm. Here, 86 parameter combinations were considered for $\Omega = 8$ rpm, and 56 parameter combinations were considered for $\Omega = 27$ rpm.

To verify the influence of the rotational speed on the phase diagram of the segregated

state, we conducted experiments at different rotational speeds, as shown in Fig. S1. We obtained qualitatively similar phase diagrams. The only apparent differences in the phase diagrams were the values of α_i and β_i. The parameters to determine the separating planes between each phase are shown in Table 1.

B. Dependence of the initial wavelength on the rotational speed

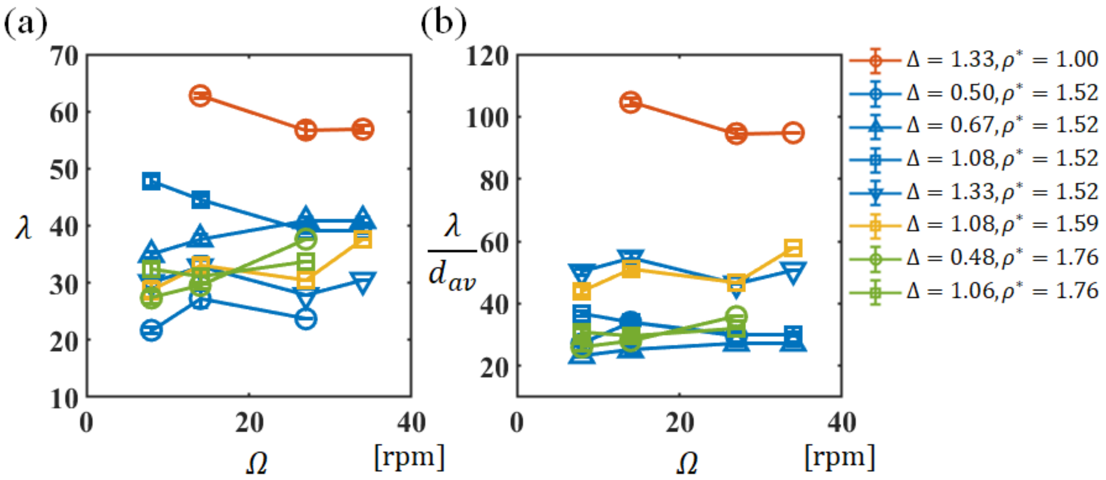

Figure S2. (a) Initial wavelength (λ) vs. rotational speed (Ω) for various particle combination. (b) Initial wavelength scaled by the average diameter (λ/d_{av}) vs. the rotational speed (Ω) with various particle combinations. The cylinder diameter is fixed at 74 mm.

The dependence of the initial wavelength on the rotational speed is shown in Fig. S2. The unscaled wavelength, λ, is plotted against Ω in Fig. S2(a) for various size differences and density ratios. No significant trends in λ with respect to changes in Ω, Δ^* or ρ^* were observed. Fig. S2(b) presents the initial wavelength scaled by the average diameter of the large and small particles as a function of Ω. The ranges of λ and λ/d_{av} increasing and decreasing with the rotational speed is wider than that of λ/d_s, as shown in Fig. 4(b). Even when plotting λ and λ/d_{av} against $D^*\,\Delta^*/\rho^*$, a positive correlation is observed, but as shown in Fig. 4(a), plotting λ/d_s against $D^*\,\Delta^*/\rho^*$ yields a pronounced linear relationship with a higher correlation coefficient.

C. Shape of the surface flow at the end wall of a cylinder

The shapes of the surface avalanche for different rotational speeds are shown in Fig. S3 for glass beads and alumina beads. Smaller grain sizes slightly distort the shape of the surface flow, but the shapes of the surface avalanche can be regarded as flat within the range of our experiments.

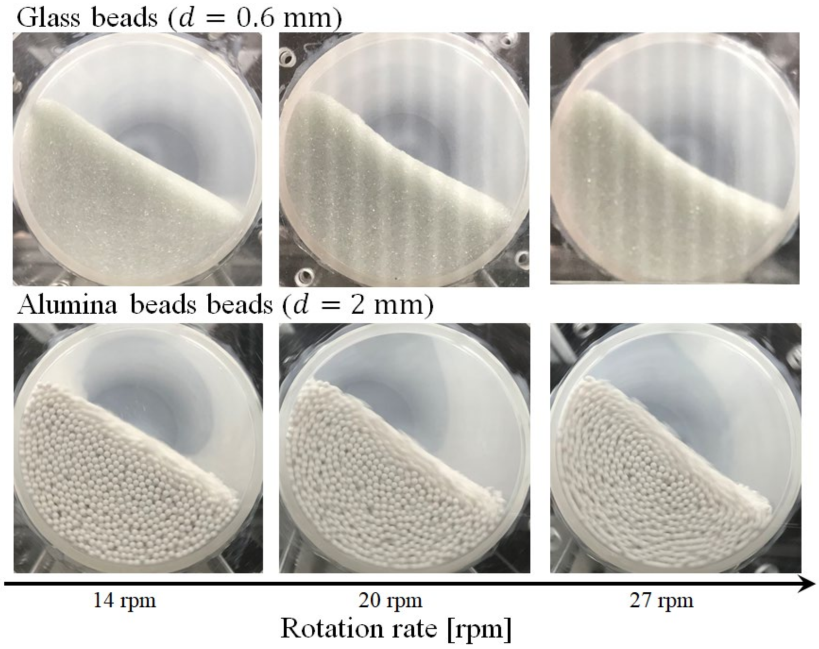

Figure S3. Shape of the surface flow from the side view.
